# Dose efficacy study of two schedules of high-dose bolus administration of interleukin 2 and interferon alpha in metastatic melanoma.

**DOI:** 10.1038/bjc.1996.463

**Published:** 1996-09

**Authors:** W. H. Kruit, C. J. Punt, S. H. Goey, P. H. de Mulder, J. W. Gratama, A. M. Eggermont, R. L. Bolhuis, G. Stoter

**Affiliations:** Department of Medical Oncology, Rotterdam Cancer Institute (Daniel den Hoed Kliniek, The Netherlands.

## Abstract

Forty-three patients with metastatic melanoma were treated with a 5 day (18 patients) and a 3 day (25 patients) schedule of high-dose IL-2 11.7 MIU m2 and IFN-alpha 3 MIU m2 i.v. by bolus administration every 8 h, repeated every 21 days for a total of three courses. The 5 day schedule resulted in a high response rate of 41% (CI 18-67%), but was accompanied by severe cardiotoxicity (41%) and central nervous system toxicity (28%). The 3 day schedule was associated with manageable toxicity, but yielded a moderate response rate of 20% (CI 7-43%).


British Journal of Cancer (1996) 74, 951-955
© 1996 Stockton Press All rights reserved 0007-0920/96 $12.00

SHORT COMMUNICATION

### Dose efficacy study of two schedules of high-dose bolus administration of interleukin 2 and interferon alpha in metastatic melanoma

WHJ Kruit[1], CJA Punt[2], SH Goey[1], PHM de Mulder[2], JW Gratama[3], AMM Eggermont[4], RLH Bolhuis[3] and G Stoter[1]

*[1]Department of Medical Oncology, Rotterdam Cancer Institute (Daniel den Hoed Kliniek) and University Hospital, Groene Hilledijk 301, 3075 EA Rotterdam, The Netherlands; [2]Department of Medical Oncology, University Hospital Nijmegen, G Grooteplein 10, 6526 GA Nijmegen, The Netherlands; Departments of [3]Medical and Tumor Immunology, [4]Surgery, Rotterdam Cancer Institute (Daniel den Hoed Kliniek) and University Hospital, Groene Hilledijk 301, 3075 EA Rotterdam, The Netherlands.*

**Summary** Forty-three patients with metastatic melanoma were treated with a 5 day (18 patients) and a 3 day (25 patients) schedule of high-dose IL-2 11.7 MIU $m^2$ and IFN-$\alpha$ 3 MIU $m^2$ i.v. by bolus administration every 8 h, repeated every 21 days for a total of three courses. The 5 day schedule resulted in a high response rate of 41% (CI 18-67%), but was accompanied by severe cardiotoxicity (41%) and central nervous system toxicity (28%). The 3 day schedule was associated with manageable toxicity, but yielded a moderate response rate of 20% (CI 7-43%).

**Keywords:** interleukin 2; interferon alpha; metastatic melanoma

The biological agents, interferon alpha (IFN-$\alpha$) and interleukin 2 (IL-2) have shown evidence of activity against metastatic melanoma. IFN-$\alpha$ produced objective tumor regression in approximately 15% of patients (Creagan *et al.*, 1990; Kirkwood, 1991). IL-2, with or without lymphokine-activated killer cells, yielded response rates from 10-25% (Rosenberg *et al.*, 1989*a*; Parkinson *et al.*, 1990; Whitehead *et al.*, 1991). With the combination of IL-2 and IFN-$\alpha$ the reported response rates varied between 0-44% (Rosenberg *et al.*, 1989*b*; Dillman *et al.*, 1993; Sparano *et al.*, 1993; Keilholz *et al.*, 1993; Kruit *et al.*, 1995). A phase I-II study with increasing dose levels of bolus IL-2 and IFN-$\alpha$ produced an objective response in 33% of patients (Rosenberg *et al.*, 1989*b*). The highest response rate of 44% was reached with a schedule of IL-2 11.7 MIU $m^{-2}$ and IFN-$\alpha$ 3 MIU $m^{-2}$ three times a day by intravenous bolus administration, 5 days per cycle. We performed a phase II study with this schedule in order to confirm these results.

### Patients and methods

### *Patient population*

The study was divided into two parts. Owing to the occurrence of severe toxicity the 5-day regimen was replaced by a 3-day schedule. All patients had histologically confirmed metastatic melanoma. Eligibility criteria included: Karnofsky performance status of at least 80, age between 18 and 70 years, no prior immunotherapy with IL-2 or IFN-$\alpha$, no metastases in the central nervous system, no cardiovascular history, normal pulmonary function, serum creatinine $\leqslant 1.25$ times the upper limit of normal, or creatinine clearance $\geqslant 50$ ml $ml^{-1}$, leucocyte count $\geqslant 4.0 \times 10^9$ $l^{-1}$, platelet count $\geqslant 100 \times 10^9$ $l^{-1}$, haemoglobin $\geqslant 9.5$ g 100 $ml^{-1}$ and normal liver function with the exception of liver function disturbances owing to metastatic disease. All patients gave informed consent and the study was carried out with ethical committee approval.

Correspondence: WHJ Kruit
Received 22 January 1996; revised 25 March 1996; accepted 28 March 1996

### *Treatment*

In part I of the study patients received recombinant IL-2 at a dose of 11.7 MIU $m^{-2}$ (Teceleukin; Hoffman La Roche, Nutley, NJ, USA) and recombinant IFN-$\alpha$ at a dose of 3 MIU $m^{-2}$ (Roferon-A, Hoffman La Roche, Basle, Switzerland), each administered as an intravenous injection over 15 min, every 8 h on days 1-5. Treatment was repeated every 21 days up to a total of three cycles. With this schedule we were confronted with severe cardiotoxicity, albeit the first 3 days of treatment were relatively well tolerated. Therefore, we decided to modify the treatment schedule, using the same daily dosages of IL-2 and IFN-$\alpha$, but now for 3 instead of 5 days.

The World Heath Organization (WHO) criteria were used to grade toxicity (WHO, 1979). Treatment was permanently discontinued if grade 3 neurotoxicity or cardiovascular toxicity occurred. In case of grade 3 hypotension, therapy was continued while giving symptomatic treatment with colloids. If volume expansion gave no improvement dopamine was added. Treatment was discontinued if the patient remained hypotensive and/or oliguric. In all other cases of grade 3 toxicity with the exception of fever, nausea/vomiting and diarrhoea, immunotherapy was discontinued until toxicity improved to grade 1 or resolved. Resumption of treatment at 50% of the previous dose was allowed in the next cycle if the grade 3 toxicity decreased to grade 1 or less. Corticosteroid administration was not allowed, except in the event of life-threatening toxicity.

The first tumour assessment was performed at 8 weeks after the start of therapy. Response evaluations were repeated every 4 weeks. Response categories were defined according to WHO criteria (WHO, 1979). The treatment results were analysed on an intent-to-treat basis. The median survival was estimated using the Kaplan-Meier method.

### *Immunological monitoring*

Absolute numbers of lymphocyte subsets and cytotoxic activities of peripheral blood mononuclear cells (PBMCs) were assessed in each treatment cycle, immediately before the first administration and 1 week later. The lymphocyte subsets defined by double-staining with CD3 and CD56, CD4 and CD8, CD16 and CD19 monoclonal antibodies were assessed by multicolour immunofluorescence and flow cytometry as described elsewhere (Gratama *et al.*, 1996). Cytotoxic activities of lymphocytes were determined in standard 3 h $^{51}$Cr-release assays. The K562 erythromyeloid leukaemia cell line and the Daudi Burkitt's lymphoma cell line were used as target cells for the assessment of natural killer (NK) and lymphokine-activated killer (LAK) activities respectively.

### Results

### *Patient characteristics*

Forty-three patients were entered in the study, 18 in part I and 25 in part II. The characteristics of the eligible patients are summarised in Table I. All eligible patients were evaluable for response and toxicity. In the 5 day regimen, one patient was ineligible because of unmeasurable disease. Two patients had previously been treated with isolated limb perfusion and one patient received radiotherapy before study entry. In the 3 day regimen, two patients were pretreated with adjuvant BCG or poly A–poly U.

### *Treatment characteristics*

Of the 17 patients in part I, nine (53%) received three cycles, three (18%) two cycles and five (29%) only one cycle. Eight patients were taken off study early; six owing to grade 3–4 toxicity, one due to rapidly progressive disease and another patient refused further treatment. The actual lymphokine doses given during the first, second and third cycle, expressed as a percentage of the planned dose, were 85%, 42% and 30% respectively.

Of the 25 patients in part II, 14 (56%), received three cycles, five (20%) two cycles and six (24%) one cycle. The lymphokine doses administered were 95%, 71% and 54% of the planned dosages respectively. Treatment was discontinued in one patient after only one single cytokine infusion owing to grade 3 toxicity. He was considered a treatment failure.

### *Treatment results*

Table II shows the results at response and survival. With the 5 day schedule the overall response rate was 41% (95%

**Table I** Patient characteristics

| | *Part I* | | *Part II* | |
|---|---|---|---|---|
| Evaluable patients | 17 | | 25 | |
| Age | | | | |
| Median | 48 | | 41 | |
| Range | 29–61 | | 20–69 | |
| Sex | | | | |
| Male | 9 | (53%) | 16 | (64%) |
| Female | 8 | (47%) | 9 | (36%) |
| Performance status (Karnofsky) | | | | |
| Median | 90 | | 90 | |
| Range | 80–100 | | 80–100 | |
| Prior therapy | | | | |
| Surgery | 17 | (100%) | 25 | (100%) |
| Immunotherapy | | | 2 | (8%) |
| Chemotherapy | 2 | (12%) | 0 | |
| Radiotherapy | 1 | (6%) | 0 | |
| Distribution of metastatic sites | | | | |
| Lung | 9 | (53%) | 10 | (40%) |
| Lymph nodes | 7 | (41%) | 17 | (68%) |
| Subcutaneous | 6 | (35%) | 12 | (48%) |
| Liver | 6 | (35%) | 9 | (36%) |
| Other (adrenal, pancreas, bone) | 8 | (47%) | 9 | (36%) |
| Number of metastatic sites | | | | |
| 1 | 6 | (35%) | 7 | (28%) |
| 2 | 4 | (24%) | 7 | (28%) |
| 3 | 4 | (24%) | 7 | (28%) |
| 4 | 2 | (12%) | 4 | (16%) |
| 5 | 1 | (6%) | | |

**Table II** Response to treatment

| | *Part I* | | *Part II* | |
|---|---|---|---|---|
| Evaluable patients | 17 | | 25 | |
| Complete response (CR) | 2 | (12%) | 0 | |
| Partial response (PR) | 5 | (29%) | 5 | (20%) |
| Overall response rate | 41% | | 20% | |
| Stable disease (SD) | 3 | (18%) | 4 | (26%) |
| Progressive disease (PD) | 7 | (41%) | 16 | (64%) |
| Median duration of response (months) | 8.6 | | 6.6 | |
| (range) | (2.0–37.5) | | (3.9–9.9) | |
| Median time to progression (months) | 3.2 | | 2.0 | |
| (range) | (0.7–37.5) | | (0.5–9.9) | |
| Median survival (months) | 10.2 | | 6.8 | |
| (range) | (2.6–37.5) | | (0.9–24+) | |

confidence interval 18–67%), including two complete responses (CRs). The overall survival was 10.2 months (range 2.6–37.5 months). With the 3 day schedule the overall response rate was 20% (95% confidence interval 7–43%). No CRs occurred. The overall survival was 6.8 months (range 0.9–24+ months).

### *Toxicity*

The adverse effects are shown in Table III. With the 5 day schedule a high incidence of severe cardiac toxicity occurred. Seven patients (41%) experienced cardiac adverse events; cardiomyopathy in four, acute cardiac arrest, myocardial

**Table III** Adverse events

| | *Part I* | | *Part II* | |
|---|---|---|---|---|
| *Adverse event* | *Grade 1/2 (%)* | *Grade 3/4 (%)* | *Grade 1/2 (%)* | *Grade 3/4 (%)* |
| Fever | 12 | 88 | 52 | 48 |
| Skin rash | 76 | 0 | 64 | 0 |
| Nausea/vomiting | 76 | 18 | 40 | 52 |
| Diarrhoea | 53 | 35 | 48 | 44 |
| Malaise | 24 | 76 | 40 | 56 |
| Weight gain | 35 | 0 | 16 | 4 |
| Hypotension | 41 | 53 | 60 | 32 |
| Cardiac | 6 | 35 | 0 | 0 |
| Dyspnoea | 53 | 24 | 28 | 8 |
| Neuropsychiatric | 41 | 29 | 32 | 4 |
| Oliguria | 18 | 18 | 20 | 12 |
| Creatinine | 47 | 0 | 12 | 0 |
| Alkaline phosphatase | 76 | 6 | 56 | 4 |
| Bilirubin | 47 | 12 | 16 | 12 |
| Transaminases | 47 | 53 | 60 | 24 |
| Anaemia | 53 | 0 | 36 | 8 |
| Thrombocytopenia | 41 | 6 | 48 | 0 |

**Figure 1** Median absolute numbers of CD56$^+$, 3$^-$ (**a**), CD3$^+$ (**b**), CD4$^+$ (**c**) and CD8$^+$ (**d**) lymphocytes in patients treated in the 5 day schedule (□) and in the 3 day schedule (■). Logarithmic scales have been used for the vertical axes in order to compress the figure. Vertical bars represent confidence limits as defined by the 5th and 95th percentile of each group. The areas between the horizontal lines represent the normal range of the different lymphocyte subsets as defined by the 5th and 95th percentiles of healthy control persons.

infarction and negative T-waves in one patient each. The details of these patients have been reported elsewhere (Kruit *et al.*, 1994). No cardiotoxicity was observed in the 3 day regimen.

Two-thirds of patients in the 5 day regimen suffered from neuropsychiatric disturbances such as agitation, disorientation, confusion and overt psychosis. With the 3-day regimen neuropsychiatric side-effects were encountered in about one-third of patients and were mild in most cases. Neurotoxicity completely resolved in all patients.

### *Immunological monitoring*

Before therapy the absolute numbers of NK lymphocytes ($CD56^+\ 3^-$ and $CD16^+$), T lymphocytes ($CD3^+$), helper/inducer T lymphocytes ($CD4^+$), cytotoxic/suppressor T lymphocytes ($CD8^+$) and B lymphocytes ($CD19^+$) were within the normal range. During therapy, the $CD56^+\ 3^-$, $CD3^+$ and $CD8^+$ lymphocyte counts gradually increased above normal, whereas the $CD4^+$ and $CD19^+$ remained within the normal range (Figure 1). The number of lymphocytes after each treatment cycle (rebound lymphocytosis) reached a higher value in the 5-day study (Figure 1, open symbols) compared with the 3-day study (closed symbols). NK and LAK cytotoxic activities of PBMCs remained within the normal range in both parts of the study, with a large variation between patients (data not shown). There was no relationship between tumour response and immune parameters.

### Discussion

From an earlier performed dose-escalating study of bolus IL-2 and IFN-α we selected the dosage schedule with the highest response rate. This treatment schedule resulted in a 41% response rate (95% CI 18–67%), which is identical to the results reported by the NCI investigators (Rosenberg *et al.*, 1989*b*).

We observed a high incidence of severe cardiotoxicity. Although myocardial toxicity was not so frequently encountered in the NCI study as here, several cases of myocardial infarction and elevation of cardiac enzyme levels in 15% of treatment courses were reported (Rosenberg *et al.*, 1989*b*; Marincola *et al.*, 1995). Also other recent literature indicates that high-dose IL-2 regimens bear a high risk of severe cardiotoxicity (Sznol *et al.*, 1992; Atkins *et al.*, 1993; Fossa *et al.*, 1993).

The second severe toxicity of the 5-day regimen consisted of neuropsychiatric disturbances. Evidence from earlier studies showed that the frequency and severity of these side-effects are dose dependent (Denicoff *et al.*, 1987; Dillman *et al.*, 1993; Sparano *et al.*, 1993; Keilholz *et al.*, 1993; Atkins *et al.*, 1993). The NCI investigators observed neurotoxicity including coma in 54% of patients (Marincola *et al.*, 1995).

Thus, the promising results on response were accompanied by unacceptable toxicities. In an attempt to maintain the high anti-tumour activity and to reduce the side-effects, we shortened the treatment duration in the second part of the study from 5 to 3 days. Indeed, the 3 day schedule was accompanied by acceptable toxicity, however, the response rate dropped to 20%. A direct comparison between the two schedules is difficult, because it was not a randomised study and the confidence intervals showed considerable overlap. The response rate and survival duration of the 3 day schedule were in the same range as observed in other studies of IL-2 and IFN-α in melanoma (Dillman *et al.*, 1993; Sparano *et al.*, 1993; Kruit *et al.*, 1995). In a recent progress report the NCI investigators observed a similar decrease in treatment results after modification of the treatment schedule (one instead of three IFN-α administrations per day), made necessary by the encountered toxicity (Marincola *et al.*, 1995). In general the response rates and survival data of combined therapy of IL-2 and IFN-α in patients with metastatic melanoma appeared not to be superior to treatment with IL-2 alone and IL-2 in combination with lymphokine-activated killer cells (Bar *et al.*, 1990; Dutcher *et al.*, 1991; Rosenberg *et al.*, 1992, 1994; Sparano *et al.*, 1993; Marincola *et al.*, 1995).

In summary, the combination of IL-2 and IFN-α has not meaningfully improved the clinical results that may be obtained with IL-2 alone. Based on the possibly additive or synergistic effects between cytotoxic agents and cytokines, biochemotherapy may be an important new treatment modality. Combinations of biological response modifiers and cytostatics have produced encouraging response rates (Richards *et al.*, 1992; Khayat *et al.*, 1993; Legha *et al.*, 1993).

**Acknowledgements**
We thank Ms P Bos for secretarial assistance and Mrs B Visser for data processing.

### References

ATKINS MB, SPARANO J, FISHER RI, WEISS GR, MARGOLIN KA, FINK KI, RUBINSTEIN L, LOUIE A, MIER JW, GUCALP R, SOSMAN JA, BOLDT DH, DOROSHOW JH, ARONSON FR AND SZNOL M. (1993). Randomized phase II trial of high-dose interleukin-2 either alone or in combination with interferon alfa-2b in advanced renal cell carcinoma. *J. Clin. Oncol.*, **11**, 661–670.

BAR MH, SZNOL M, ATKINS MB, CIOBANU N, MICETICH KC, BOLDT DH, MARGOLIN KA, ARONSON FR, RAYNER AA, HAWKINS MJ, MIER JW, PAIETTA E, FISHER RI, WEISS GR AND DOROSHOW JH. (1990). Metastatic malignant melanoma treated with combined bolus and continuous infusion interleukin-2 and lymphokine-activated killer cells. *J. Clin. Oncol.*, **8**, 1138–1147.

CREAGAN ET, SCHAID DJ, AHMANN DL AND FRYTAK S. (1990). Disseminated malignant melanoma and recombinant interferon: analysis of seven consecutive phase II investigations. *J. Invest. Dermatol.*, **95**, 118S–192S.

DENICOFF KD, RUBINOW DR, PAPA MZ, SIMPSON C, SEIPP CA, LOTZE MT, CHANG AE, ROSENSTEIN D AND ROSENBERG SA. (1987). The neuro-psychiatric effects of treatment with interleukin-2 and lymphokine activated killer cells. *Ann. Intern. Med.*, **107**, 293–300.

DILLMAN RO, CHURCH C, OLDHAM RK, WEST WH, SCHWARTZBERG L AND BIRCH R. (1993). Inpatient continuous-infusion interleukin-2 in 788 patients with cancer. The national biotherapy study group experience. *Cancer*, **71**, 2358–2370.

DUTCHER JP, GAYNOR ER, BOLDT DH, DOROSHOW JH, BAR MH, SZNOL M, MIER J, SPARANO J, FISHER RI, WEISS G, MARGOLIN K, ARONSON FR, HAWKINS M AND ATKINS M. (1991). A phase II study of high-dose continuous infusion interleukin-2 with lymphokine-activated killer cells in patients with metastatic melanoma. *J. Clin. Oncol.*, **9**, 641–648.

FOSSÅ SD, AUNE H, BAGGERUD E, GRANERUD T, HEILO A AND THEODORSEN L. (1993). Continuous intravenous interleukin-2 infusion and subcutaneous interferon-α in metastatic renal cell carcinoma. *Eur. J. Cancer*, **29A**, 1313–1315.

GRATAMA JW, SCHMITZ PIM, GOEY SH, LAMERS CHJ, STOTER G AND BOLHUIS RLH. (1996). Modulation of immune parameters in patients with metastatic renal cell cancer receiving combination immunotherapy (IL-2, IFNα and autologous IL-2 activated lymphocytes). *Int. J. Cancer*, **65**, 152–160.

KEILHOLZ U, SCHEIBENBOGEN C, TILGEN W, BERGMANN L, WEIDMANN E, SEITHER E, RICHTER M, BRADO B, MITROU PS AND HUNSTEIN W. (1993). Interferon-α and interleukin-2 in the treatment of metastatic malanoma. Comparison of two phase II trials. *Cancer*, **72**, 607–614.

KHAYAT D, BOREL C, TOURANI JM, BENHAMMOUDA A, ANTOINE E, RIXE O, VUILLEMIN E, BAZEX PA, THILL L, FRANKS R, AUCLERC G, SOUBRANE C, BANZET P AND WEIL M. (1993). Sequential chemoimmunotherapy with cisplatin, interleukin-2, and interferon alfa-2a for metastatic melanoma. *J. Clin. Oncol.*, **11**, 2173–2180.

KIRKWOOD JM. (1991). Studies of interferons in the therapy of melanoma. *Semin. Oncol.*, **18**, (suppl. 7) 83–89.

KRUIT WHJ, PUNT CJA, GOEY SH, DE MULDER PH, VAN HOOGENHUIJZE DC, HENZEN-LOGMANS SC AND STOTER G. (1994). Cardiotoxicity as a dose-limiting factor in a schedule of high dose bolus therapy with interleukin-2 and alpha-interferon. *Cancer*, **74**, 1850–1856.

KRUIT WHJ, GOEY SH, CALABRESI F, LINDEMANN A, STAHEL RA, POLIWODA H, OSTERWALDER B AND STOTER G. (1995). Final report of a phase II study of interleukin-2 and interferon-α in patients with metastatic melanoma. *Br. J. Cancer*, **71**, 1319–1321.

LEGHA SS AND BUZAID AC. (1993). Role of recombinant interleukin-2 in combination with interferon-alfa and chemotherapy in the treatment of advanced melanoma. *Sem. Oncol.*, **20**, (suppl), 27–32.

MARINCOLA FM, WHITE DE, WISE AP AND ROSENBERG SA. (1995). Combination therapy with interferon alfa-2a and interleukin-2 for the treatment of metastatic cancer. *J. Clin. Oncol.*, **13**, 1110–1122.

PARKINSON DR, ABRAMS JS, WIERNIK PH, RAYNER AA, MARGOLIN KA, VAN ECHO DA, SZNOL M, DUTCHER JP, ARONSON FR, DOROSHOW JH, ATKINS MB AND HAWKINS MJ. (1990). Interleukin-2 therapy in patients with metastatic malignant melanoma: a phase II study. *J. Clin. Oncol.*, **8**, 1650–1656.

RICHARDS JM, MEHTA N, RAMMING K AND SKOSEY P. (1992). Sequential chemoimmunotherapy in the treatment of metastatic melanoma. *J. Clin. Oncol.*, **10**, 1338–1343.

ROSENBERG SA, LOTZE MT, YANG JC, AEBERSOLD PM, LINEHAN WM, SEIPP CA AND WHITE DE. (1989*a*). Experience with the use of high-dose interleukin-2 in the treatment of 652 cancer patients. *Ann. Surg.*, **210**, 474–485.

ROSENBERG SA, LOTZE MT, YANG JC, LINEHAN WM, SEIPP CA, CALABRO S, KARP SE, SHERRY RM, STEINBERG S AND WHITE DE. (1989*b*). Combination therapy with interleukin-2 and α-interferon for the treatment of patients with advanced cancer. *J. Clin. Oncol.*, **7**, 1863–1874.

ROSENBERG SA, LOTZE MT, YANG JC, TOPALIAN S, CHANG AE, SCHWARTZENTRUBER DJ, AEBERSOLD P, LEITMAN S, LINEHAN WM, SEIPP CA, WHITE DE AND STEINBERG SM. (1992). Prospective randomized trial of high-dose interleukin-2 alone or in conjunction with lymphokine-activated killer cells for the treatment of patients with advanced cancer. *J. Natl Cancer Inst.*, **85**, 622–632.

ROSENBERG SA, YANG JC, TOPALIAN SL, SCHWARTZENTRUBER DJ, WEBER JS, PARKINSON DR, SEIPP CA, EINHORN JH AND WHITE DE. (1994). Treatment of 283 consecutive patients with metastatic melanoma or renal cell cancer using high-dose bolus interleukin-2. *JAMA*, **271**, 907–913.

SPARANO JA, FISHER RI, SUNDERLAND M, MARGOLIN K, ERNEST ML, SZNOL M, ATKINS MB, DUTCHER JP, MICETICH KC, WEISS GR, DOROSHOW JH, ARONSON FR, RUBINSTEIN LV AND MIER JW. (1993). Randomized phase III trial of treatment with high-dose interleukin-2 either alone or in combination with interferon alfa-2a in patients with advanced melanoma. *J. Clin. Oncol.*, **11**, 1969–1977.

SZNOL M, CLARK JW, SMITH JW, STEIS RG, URBA WJ, RUBINSTEIN LV, VANDERMOLEN LA, JANIK J, SHARFMAN WH, FENTON RG, CREEKMOORE SP, KREMERS P, CONLON K, HURSEY J, BEVERIDGE J AND LONGO DL. (1992). Pilot study of interleukin-2 and lymphokine-activated killer cells combined with immunomodulatory doses of chemotherapy and sequenced with interferon alfa-2a in patients with metastatic melanoma and renal cell carcinoma. *J. Natl Cancer Inst.*, **84**, 929–937.

WHITEHEAD RP, KOPECKY KJ, SAMSON MK, COSTANZI JJ, NATALE RB, FEUN LG, HERSH EM AND RINEHART JJ. (1991). Phase II study of intravenous bolus recombinant interleukin-2 in advanced malignant melanoma. *J. Natl Cancer Inst.*, **83**, 1250–1252.

WORLD HEALTH ORGANIZATION. (1979). *WHO Handbook for Reporting Results of Cancer Treatment*. WHO: Geneva.


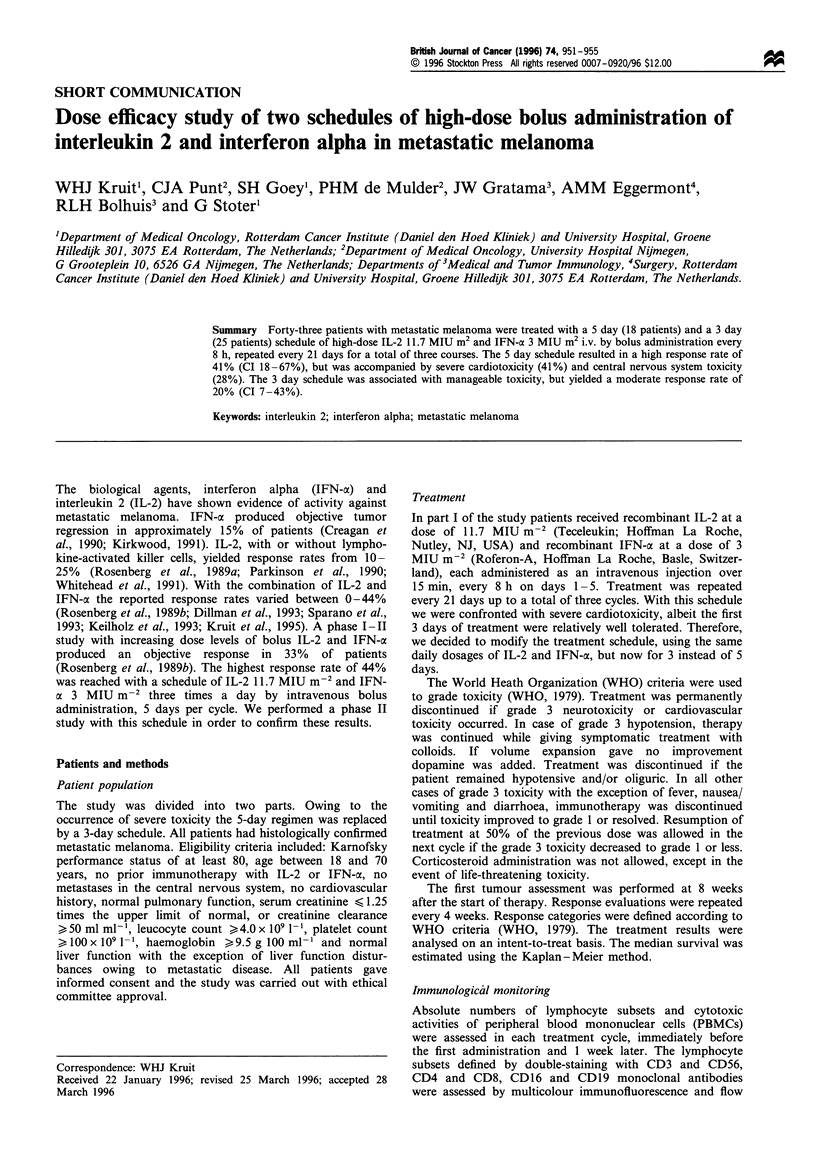

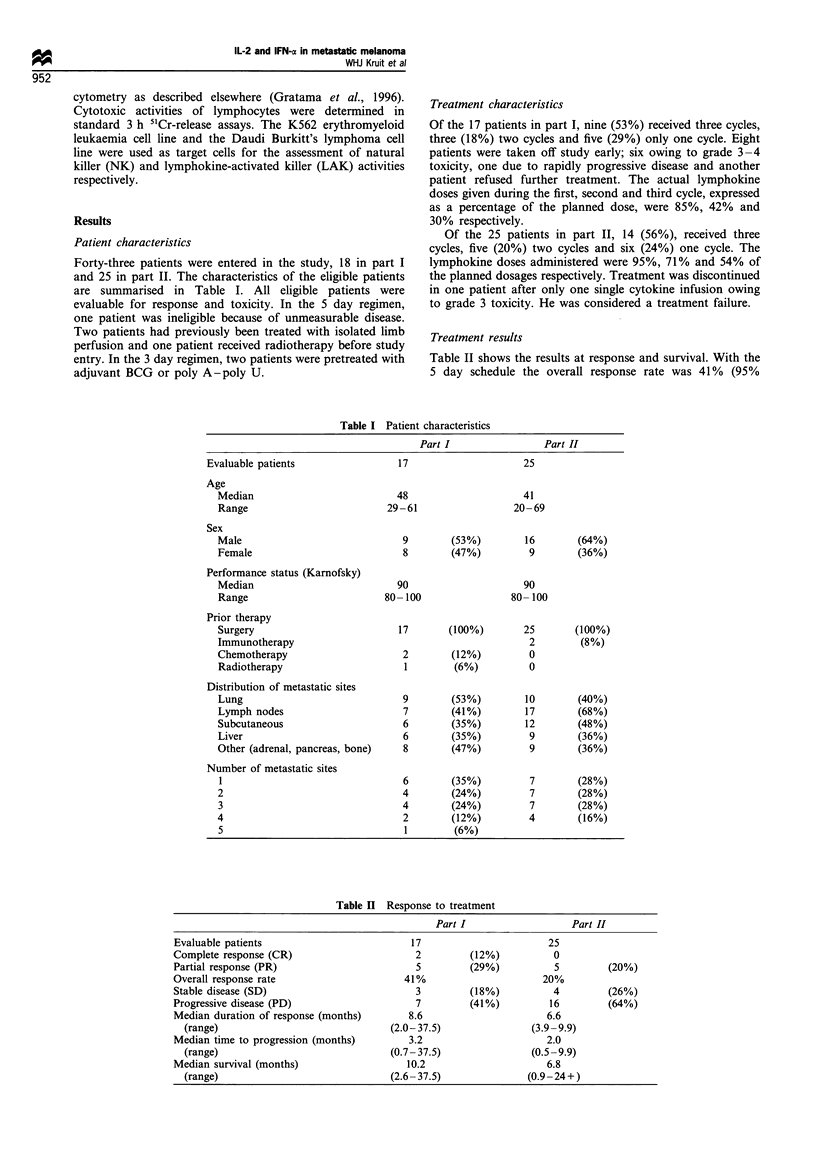

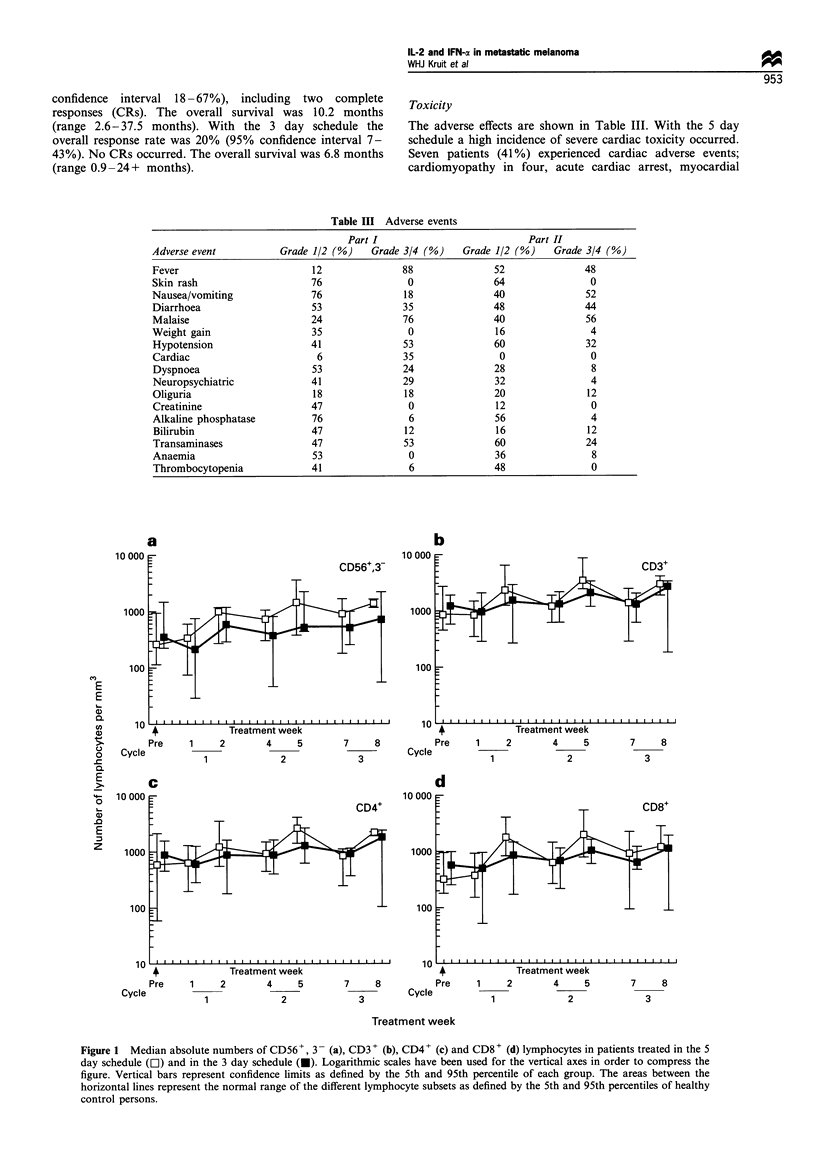

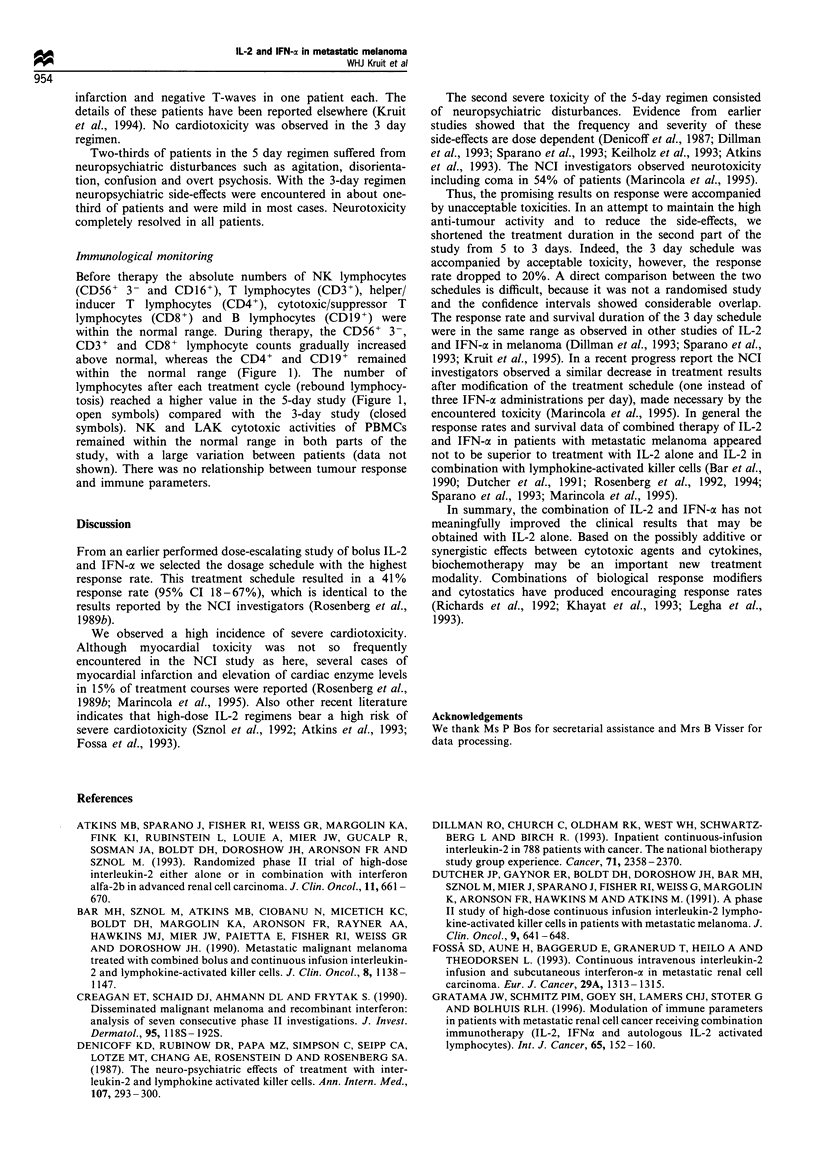

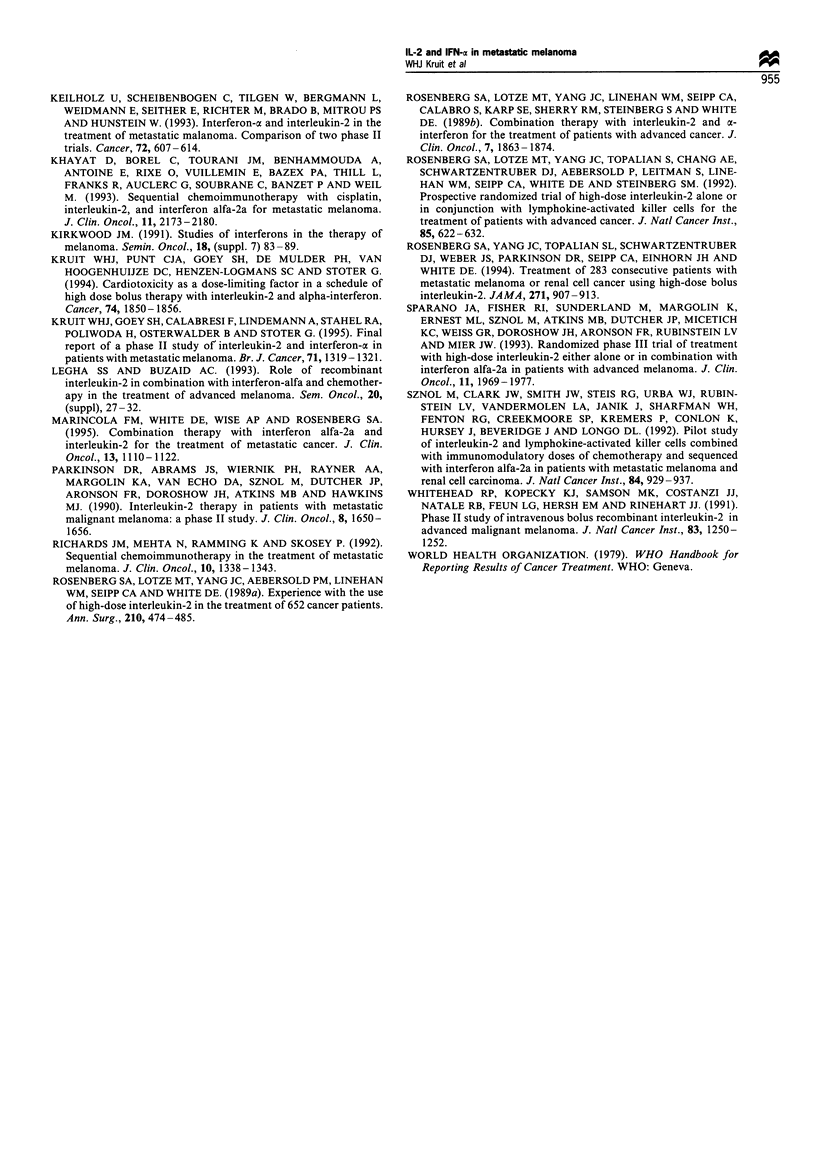


## References

[OCR_00546] Atkins M. B., Sparano J., Fisher R. I., Weiss G. R., Margolin K. A., Fink K. I., Rubinstein L., Louie A., Mier J. W., Gucalp R. (1993). Randomized phase II trial of high-dose interleukin-2 either alone or in combination with interferon alfa-2b in advanced renal cell carcinoma.. J Clin Oncol.

[OCR_00557] Bar M. H., Sznol M., Atkins M. B., Ciobanu N., Micetich K. C., Boldt D. H., Margolin K. A., Aronson F. R., Rayner A. A., Hawkins M. J. (1990). Metastatic malignant melanoma treated with combined bolus and continuous infusion interleukin-2 and lymphokine-activated killer cells.. J Clin Oncol.

[OCR_00564] Creagan E. T., Schaid D. J., Ahmann D. L., Frytak S. (1990). Disseminated malignant melanoma and recombinant interferon: analysis of seven consecutive phase II investigations.. J Invest Dermatol.

[OCR_00571] Denicoff K. D., Rubinow D. R., Papa M. Z., Simpson C., Seipp C. A., Lotze M. T., Chang A. E., Rosenstein D., Rosenberg S. A. (1987). The neuropsychiatric effects of treatment with interleukin-2 and lymphokine-activated killer cells.. Ann Intern Med.

[OCR_00575] Dillman R. O., Church C., Oldham R. K., West W. H., Schwartzberg L., Birch R. (1993). Inpatient continuous-infusion interleukin-2 in 788 patients with cancer. The National Biotherapy Study Group experience.. Cancer.

[OCR_00581] Dutcher J. P., Gaynor E. R., Boldt D. H., Doroshow J. H., Bar M. H., Sznol M., Mier J., Sparano J., Fisher R. I., Weiss G. (1991). A phase II study of high-dose continuous infusion interleukin-2 with lymphokine-activated killer cells in patients with metastatic melanoma.. J Clin Oncol.

[OCR_00592] Fosså S. D., Aune H., Baggerud E., Granerud T., Heilo A., Theodorsen L. (1993). Continuous intravenous interleukin-2 infusion and subcutaneous interferon-alpha in metastatic renal cell carcinoma.. Eur J Cancer.

[OCR_00602] Gratama J. W., Schmitz P. I., Goey S. H., Lamers C. H., Stoter G., Bolhuis R. L. (1996). Modulation of immune parameters in patients with metastatic renal-cell cancer receiving combination immunotherapy (IL-2, IFN alpha and autologous IL-2-activated lymphocytes).. Int J Cancer.

[OCR_00611] Keilholz U., Scheibenbogen C., Tilgen W., Bergmann L., Weidmann E., Seither E., Richter M., Brado B., Mitrou P. S., Hunstein W. (1993). Interferon-alpha and interleukin-2 in the treatment of metastatic melanoma. Comparison of two phase II trials.. Cancer.

[OCR_00618] Khayat D., Borel C., Tourani J. M., Benhammouda A., Antoine E., Rixe O., Vuillemin E., Bazex P. A., Thill L., Franks R. (1993). Sequential chemoimmunotherapy with cisplatin, interleukin-2, and interferon alfa-2a for metastatic melanoma.. J Clin Oncol.

[OCR_00637] Kruit W. H., Goey S. H., Calabresi F., Lindemann A., Stahel R. A., Poliwoda H., Osterwalder B., Stoter G. (1995). Final report of a phase II study of interleukin 2 and interferon alpha in patients with metastatic melanoma.. Br J Cancer.

[OCR_00641] Legha S. S., Buzaid A. C. (1993). Role of recombinant interleukin-2 in combination with interferon-alfa and chemotherapy in the treatment of advanced melanoma.. Semin Oncol.

[OCR_00645] Marincola F. M., White D. E., Wise A. P., Rosenberg S. A. (1995). Combination therapy with interferon alfa-2a and interleukin-2 for the treatment of metastatic cancer.. J Clin Oncol.

[OCR_00654] Parkinson D. R., Abrams J. S., Wiernik P. H., Rayner A. A., Margolin K. A., Van Echo D. A., Sznol M., Dutcher J. P., Aronson F. R., Doroshow J. H. (1990). Interleukin-2 therapy in patients with metastatic malignant melanoma: a phase II study.. J Clin Oncol.

[OCR_00661] Richards J. M., Mehta N., Ramming K., Skosey P. (1992). Sequential chemoimmunotherapy in the treatment of metastatic melanoma.. J Clin Oncol.

[OCR_00667] Rosenberg S. A., Lotze M. T., Yang J. C., Aebersold P. M., Linehan W. M., Seipp C. A., White D. E. (1989). Experience with the use of high-dose interleukin-2 in the treatment of 652 cancer patients.. Ann Surg.

[OCR_00670] Rosenberg S. A., Lotze M. T., Yang J. C., Linehan W. M., Seipp C., Calabro S., Karp S. E., Sherry R. M., Steinberg S., White D. E. (1989). Combination therapy with interleukin-2 and alpha-interferon for the treatment of patients with advanced cancer.. J Clin Oncol.

[OCR_00677] Rosenberg S. A., Lotze M. T., Yang J. C., Topalian S. L., Chang A. E., Schwartzentruber D. J., Aebersold P., Leitman S., Linehan W. M., Seipp C. A. (1993). Prospective randomized trial of high-dose interleukin-2 alone or in conjunction with lymphokine-activated killer cells for the treatment of patients with advanced cancer.. J Natl Cancer Inst.

[OCR_00686] Rosenberg S. A., Yang J. C., Topalian S. L., Schwartzentruber D. J., Weber J. S., Parkinson D. R., Seipp C. A., Einhorn J. H., White D. E. (1994). Treatment of 283 consecutive patients with metastatic melanoma or renal cell cancer using high-dose bolus interleukin 2.. JAMA.

[OCR_00696] Sparano J. A., Fisher R. I., Sunderland M., Margolin K., Ernest M. L., Sznol M., Atkins M. B., Dutcher J. P., Micetich K. C., Weiss G. R. (1993). Randomized phase III trial of treatment with high-dose interleukin-2 either alone or in combination with interferon alfa-2a in patients with advanced melanoma.. J Clin Oncol.

[OCR_00704] Sznol M., Clark J. W., Smith J. W., Steis R. G., Urba W. J., Rubinstein L. V., VanderMolen L. A., Janik J., Sharfman W. H., Fenton R. G. (1992). Pilot study of interleukin-2 and lymphokine-activated killer cells combined with immunomodulatory doses of chemotherapy and sequenced with interferon alfa-2a in patients with metastatic melanoma and renal cell carcinoma.. J Natl Cancer Inst.

[OCR_00712] Whitehead R. P., Kopecky K. J., Samson M. K., Costanzi J. J., Natale R. B., Feun L. G., Hersh E. M., Rinehart J. J. (1991). Phase II study of intravenous bolus recombinant interleukin-2 in advanced malignant melanoma: Southwest Oncology Group study.. J Natl Cancer Inst.

